# Developing a database of systematic reviews of animal studies

**DOI:** 10.1016/j.yrtph.2021.104940

**Published:** 2021-05-06

**Authors:** Miranda W. Langendam, Kristen Magnuson, Ashley R. Williams, Vickie R. Walker, Kembra L. Howdeshell, Andrew A. Rooney, Carlijn R. Hooijmans

**Affiliations:** a Amsterdam University Medical Centres, University of Amsterdam, Department of Epidemiology and Data Science, Amsterdam Public Health Research Institute, the Netherlands; b ICF, Durham, NC, USA; c Division of the National Toxicology Program, National Institute of Environmental Health Sciences, National Institutes of Health, Research Triangle Park, NC, USA; d Systematic Review Centre for Laboratory Animal Experimentation (SYRCLE), Department for Health Evidence, Radboud Institute for Health Sciences, Radboud University Medical Center, Nijmegen, the Netherlands

**Keywords:** Database, Systematic reviews, Animal studies

## Abstract

Systematic reviews (SRs) are common practice in clinical and public health research, but less common in non-human animal research. Systematic reviews of animal studies can be valuable to inform clinical research, to evaluate the need for further animal experiments on a given topic, and to assess the hazard of an environmental exposure in the evaluation of toxicological studies. In the last 10 years, there has been an increase in the number of SRs of animal research, as well as several publications with detailed guidance on how to perform high-quality systematic reviews of experimental animal studies. In order to evaluate current analytical approaches used in SRs of animal studies, easily identify all systematic reviews on a specific topic, and subsequently the original animal studies and their results and promote awareness and understanding of these emerging approaches, we compiled a database of SRs of animal studies. The database was developed using a rigorous, systematic approach and covers a broad range of research fields: preclinical research, toxicology, environmental health, and veterinary medicine. The database currently includes 3113 SRs of animal studies (search date June 2019). In addition to bibliographical information, data on whether or not a risk of bias assessment and meta-analysis were conducted were extracted. For future users, the search features of the database provide users with a platform to identify and select SRs with a particular characteristic for export to Microsoft Word or Microsoft Excel. From there, users may perform additional data extraction to meet their research needs. The database is freely available at www.Mendeley.com (link). The database provides methodologists a comprehensive source that can be used to explore and advance the current methodology applied to SRs of animal studies, and can help researchers to easily identify all systematic reviews on a specific topic, and subsequently the original animal studies and their results and avoid duplication and unnecessary animal research.

## 1. Introduction

Well conducted systematic reviews (SRs) follow prespecified methods for identifying, selecting, appraising and synthesizing available evidence to answer a specific research question in a way that maximizes transparency and minimizes bias in the review process. Systematic reviews are common practice in clinical (e.g., Cochrane reviews) and public health research (e.g., Agency for Healthcare Research and Quality [AHRQ] reviews), but are less common in non-human animal research (Korevaar et al., 2011; van Luijk et al., 2014). This type of review of animal studies can be quite valuable to inform clinical research, to evaluate the need for further animal experiments on a given topic, and to assess the hazard of an environmental exposure in the evaluation of toxicological studies (de Vries et al., 2014a; Sena et al., 2014; Wikoff and G.W. M, 2018).

In the last 10 years, there has been an increase in the number of systematic reviews of animal research, as well as several publications with detailed guidance on how to perform high-quality systematic reviews of experimental animal studies (Korevaar et al., 2011; Sena et al., 2014; Hooijmans et al., 2010, 2014a, 2014b, 2018; de Vries et al., 2014b, 2015; Leenaars et al., 2012; Vesterinen et al., 2013). The Collaborative Approach to Meta-Analysis and Review of Animal Data from Experimental Studies (CAMARADES^1^) and the Systematic Review Center for Laboratory animal Experimentation (SYRCLE^2^) are two research groups that have moved the field forward in developing methods and providing training and methodological support for the assessment of preclinical animal studies using systematic review approaches. An important goal of preclinical animal studies is to collect data in support of the safety of new treatments (e.g., drugs, gene therapy) and to assess the safety profile of medical devices or diagnostic tools). Preclinical studies aim to unravel potential pathophysiological mechanisms and pathways of the treatment, device or tool under development, and they are required before the start of clinical trials in humans.

The fields of toxicology and environmental health utilize experimental animal studies as part of an overall testing strategy that relies on modeling, in vitro assays and in vivo assays to assess the toxicity of chemicals. In addition, animal studies are a critical component of toxicological risk assessment, which determine safe exposure levels for chemicals based on these studies and other types of evidence. Toxicological SRs face specific challenges, such as the integration of evidence across diverse study designs and multiple evidence streams (e.g., human, animal, and in vitro studies) in reaching conclusion on potential health effects for a given chemical (Hoffmann et al., 2017). Organizations like the National Toxicology Program’s (NTP’s) Office of Health Assessment and Translation (OHAT), the Evidence-based Toxicology Collaboration, and the European Food and Safety Agency have been instrumental in developing SR methods that include the appraisal of study quality of animal studies and guidance for integrating evidence across evidence streams (Hoffmann et al., 2017; Woodruff and Sutton, 2014).

In order to evaluate current analytical approaches used in SRs of animal studies and promote awareness and understanding of these emerging approaches, we compiled a database of SRs of animal studies (i.e., reviews that focused exclusively on non-human animal research, or reviews that included animal studies along with human studies). This database was developed using a rigorous, systematic approach and it covers a broad range of research fields: preclinical research, toxicology, environmental health, and veterinary medicine. Since SRs are not always labelled as such in search databases such as Pubmed, in this paper a SR qualifies as such when the title or abstract states the aim to systematically review the literature, eligibility criteria for the primary studies are reported, search terms are specified, and the search is performed in at least one specified database/electronic source.

The database can be used in different ways and serves several purposes. First, the database can be used to avoid duplication of effort and, thus, reduce research waste. The database makes it easy for researchers to identify published SRs of animal studies, which allows them to identify whether their research question has been previously addressed. For example, the implementation of SR of animal studies has led to a 35% reduction in animal use in experiments at the Radboud University in the Netherlands (Cochrane, 2017). In addition, this database provides an opportunity for researchers to investigate questions, such as: how many animal SRs are published on a given intervention or chemical exposure? how has the number of studies included in SRs evolved over time? and are there publication trends related to the chemical or substance under review (e.g., how have the number of SRs on arsenic fluctuated over time)? This database compliments other efforts to avoid duplication of research efforts such as PROSPERO,^3^ an international database of prospectively registered SRs, which began publishing the protocols for SRs of animal studies in December 2017. Thus, the current database provides information on published SRs from a time before protocol registration or publication of SR protocols for animal study SRs was possible.

Second, the database facilitates easily identifying all systematic reviews on a specific topic, and subsequently the original animal studies and their results. For example, when someone is interested in the effects of omega-3 fatty acids or a specific chemical in laboratory animals, they can use the database (search by topic) to easily retrieve all systematic reviews about this topic. This is especially valuable since Identifying systematic reviews in search databases such as PubMed, web of science and Embase is very challenging and time consuming, due to the large numbers of references that need to be screened when searching databases. In addition, not all systematic reviews and meta analyses are labelled as such in these search engines. Moreover, by identifying the SRs in the field of interest, researchers obtain insight in the individual animal studies and their results as well.

Third, the database can be used for the creation of evidence maps, usually designed as interactive figures of study characteristics; for example, an evidence map might identify and categorize studies (e.g., human studies sorted by study design and animal studies sorted by species or strain) on all of the health effects associated with a particular chemical exposure, or the experimental animal models used to investigate a particular disease. Evidence maps provide insight into topics with abundant evidence, and those for which aggregated evidence is lacking, which will be useful in developing future research agendas.

Lastly, this database can be used for conducting a meta-epidemiological analysis on the determinants of methodological quality of SRs and appropriateness of meta-analysis (MA) of animal studies. This resource of currently used methods will enable researchers to focus efforts on where and how to improve the methodology of evidence synthesis in these research fields, particularly in developing new MA approaches. Challenges facing MAs of animals studies include the selection of the effect measure, given the diversity in study designs and animal species studied, and accounting for heterogeneity (cross-species sensitivity, different ways of measuring an endpoint, differences in the age or duration of exposure, age at testing, and variations in the presentation of results). For example, the database has been used to obtain an overview of the key characteristics of published meta-analyses of human health related animal studies, but also to gain insight into the rationale behind the choice of a specific continuous effect size measures as described in a separate publication in this issue of regulatory toxicology and pharmacology (Hooijmans et al., 2020). Because the database covers all fields in which animal studies are performed, it also can promote harmonization of SR methods across fields and thereby enhance collaboration.

This paper introduces the database by describing its development, main features and temporal trends in published animal SRs.

## 2. Methods

### 2.1. Definition of SR

In this manuscript, a SR qualifies as such when the title or abstract states the aim to systematically review the literature, eligibility criteria for the primary studies are reported, search terms are specified, and the search is performed in at least one specified database/electronic source.

It is important to note here that we did not appraise the methodological quality of the SRs, but applied a minimum set of criteria for the definition of SRs.

### 2.2. Information sources

To identify all published SRs of animal studies, three databases were searched from their inception through June 18, 2019: MEDLINE (via PubMed), Embase (via Ovid), and Web of Science 2019. An additional clean up step was applied to the Web of Science search results to identify the records with search terms in the title and abstract versus only in the keyword field (see details in Appendix A).

### 2.3. Search strategy

The search strategy combined search terms for SRs with search filters for animal studies (de Vries et al., 2014b; Hooijmans et al., 2010). For the search component systematic reviews we included besides various terminology for systematic reviews such as meta-analyses, systematic literature review, comprehensive literature review, systematic survey OR systematic overview, also terminology that referred to a systematic or comprehensive literature search in combination with either database names or the mentioning of selection criteria.The search filters for animal studies (de Vries et al., 2011, 2014b; Hooijmans et al., 2010) focused on species that are considered experimental animal models by European legislation. The PubMed search filter also includes the most frequently used invertebrate species, such as nematodes and fruit flies. Appendix A presents the detailed search strategy for each information source. The search was conducted December 22, 2015; update search: January 10, 2017; update search: February 13, 2018; update search: June 18, 2019.

### 2.4. Eligibility criteria

The references (also called records) included in the animal studies SR database meet the following eligibility criteria:

1. The reference aims to systematically review the literature. The title or abstract states this aim using terminology such as “literature review,” “literature overview,” “systematic review,” “systematic survey,” or “meta-analysis.”
2. The reference summarizes the results of studies in laboratory or experimental animals to investigate human or animal health.
3. The reference reports the eligibility criteria for the primary studies, specifies search terms, and the search is performed in at least one specified database/electronic source (e.g., PubMed).
4. A full text version of the reference is publicly available. In this manuscript this means the reference is available online or in print from open access sources, journal subscriptions, or inter-library loan requests.

There were no restrictions in language or publication date.

### 2.5. Study selection, data collection, and database features

The full methodological details are described in Appendix B. In brief, title-and-abstract screening of the initial literature search results were assisted by the Document Classification and Topic Extraction Resource (DoCTER; ICF, Durham, North Carolina, USA), a machine-learning software that prioritized the references by relevance based on a training set of relevant animal SRs (Varghese et al., 2018a).

DoCTER is publicly available at https://www.icf-docter.com/, and the methods have been peer reviewed previously (Varghese et al., 2018b, 2019)All references predicted by DoCTER as not relevant were excluded. The remaining references were manually screened for relevance by title and abstract by two independent screeners. A third screener resolved conflicts as necessary. References identified as relevant or possibly relevant in title-and-abstract screening were screened for relevance in full-text screening, which was conducted manually by two independent screeners. A third screener resolved conflicts in full-text screening, as necessary. Subsequent literature search updates were manually screened at the title-and-abstract and full-text levels following the same methods. Appendix B contains detailed methods for the study selection and data collection process.

For each reference, a single person extracted data elements and entered them into a Microsoft Access database (Redmond, WA). Data elements included author, publication year, title, abstract, citation, total number of sources (e.g., databases) searched, names of sources searched, performance of meta-analysis (yes/no), and use of study quality assessment (yes/no).

We defined quality assessment when the authors of the review used a formal quality assessment tool or provided an explanation of the criteria used to evaluate the study. Meta-analysis was defined as an effort to combine the included studies in a quantitative assessment.

The database includes a search interface that allows users to view all references (i.e., records) included in the database or a subset of records based on the selection of specific data elements. Users can also perform keyword searches to refine the record selection to a specific topic. Appendix C describes the database features and provides an example of how to use the database to find SRs on a particular chemical (see Box C-1 in Appendix C). This appendix can also be used when actually using the database at www.Mendeley.com.

## 3. Results

### 3.1. Search and study selection

**Fig. 1.**
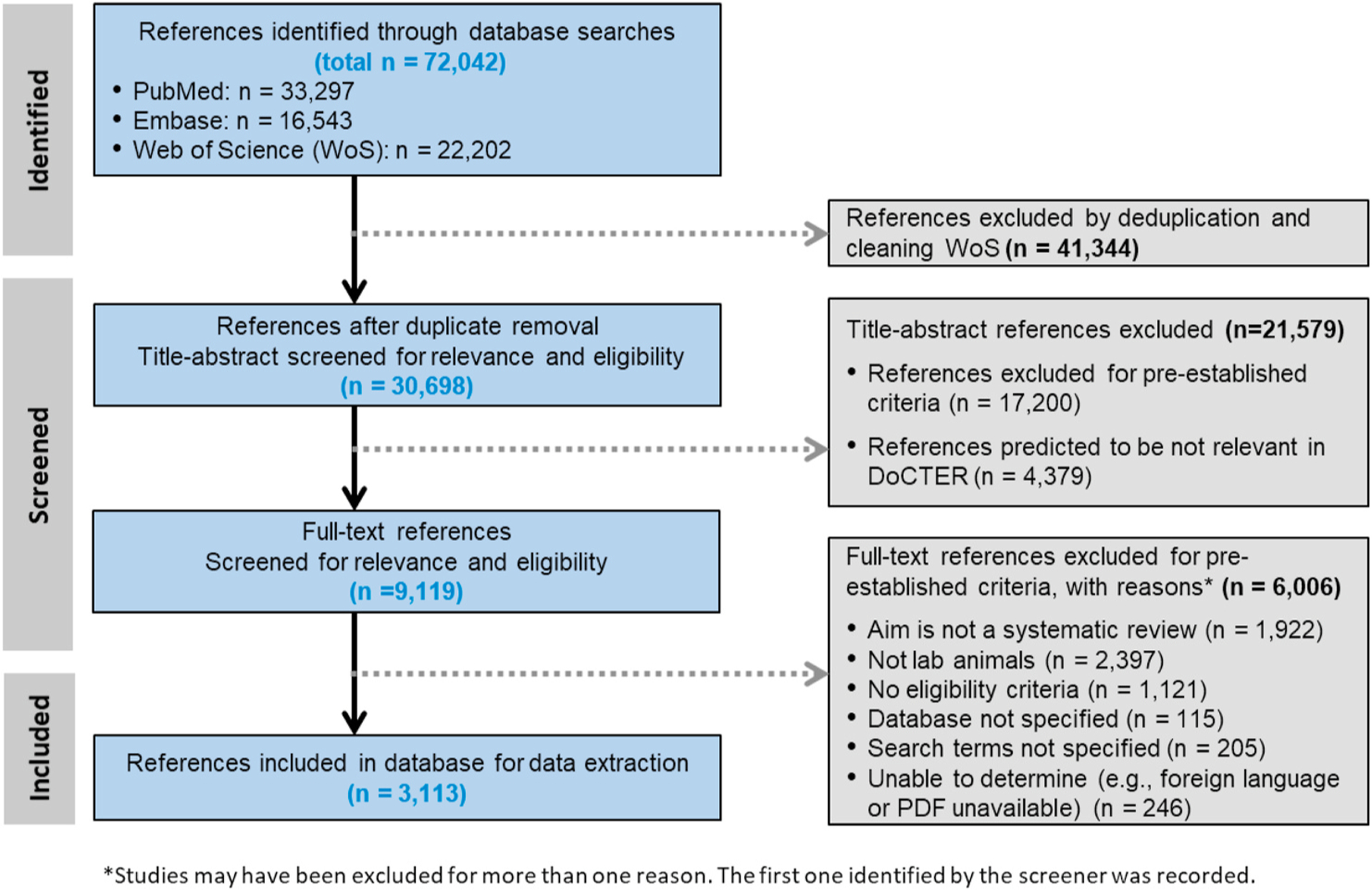
Study selection diagram.

The search of electronic databases for animal study SRs resulted in 72,042 references. After de-duplication of the references and cleaning up the Web of Science results to include only references with an animal term in the title and/or abstract, 30,691 references were screened for relevance assisted by prioritization by title-and-abstract using machine-learning in DoCTER (conducted on the initial search results), then manual screening of title and abstract followed by manual screening of full text (conducted on the initial search results and updates). A total of 3113 references met the inclusion criteria through full-text screening and were included in the database (Fig. 1).

### 3.2. Availability of the database

The database, titled: “Database of Animal Systematic Review Publications” is freely available at www.data.mendeley.com (https://data/.mendeley.com/drafts/6fr3nw5mpc). Mendeley data is a platform where you can make your data openly available. It contains the actual database, the search strategies and eligibility criteria used, and the steps to reproduce in case scientists want to use and update the database.

### 3.3. Characteristics of systematic reviews of animal studies and trends over time

**Fig. 2.**
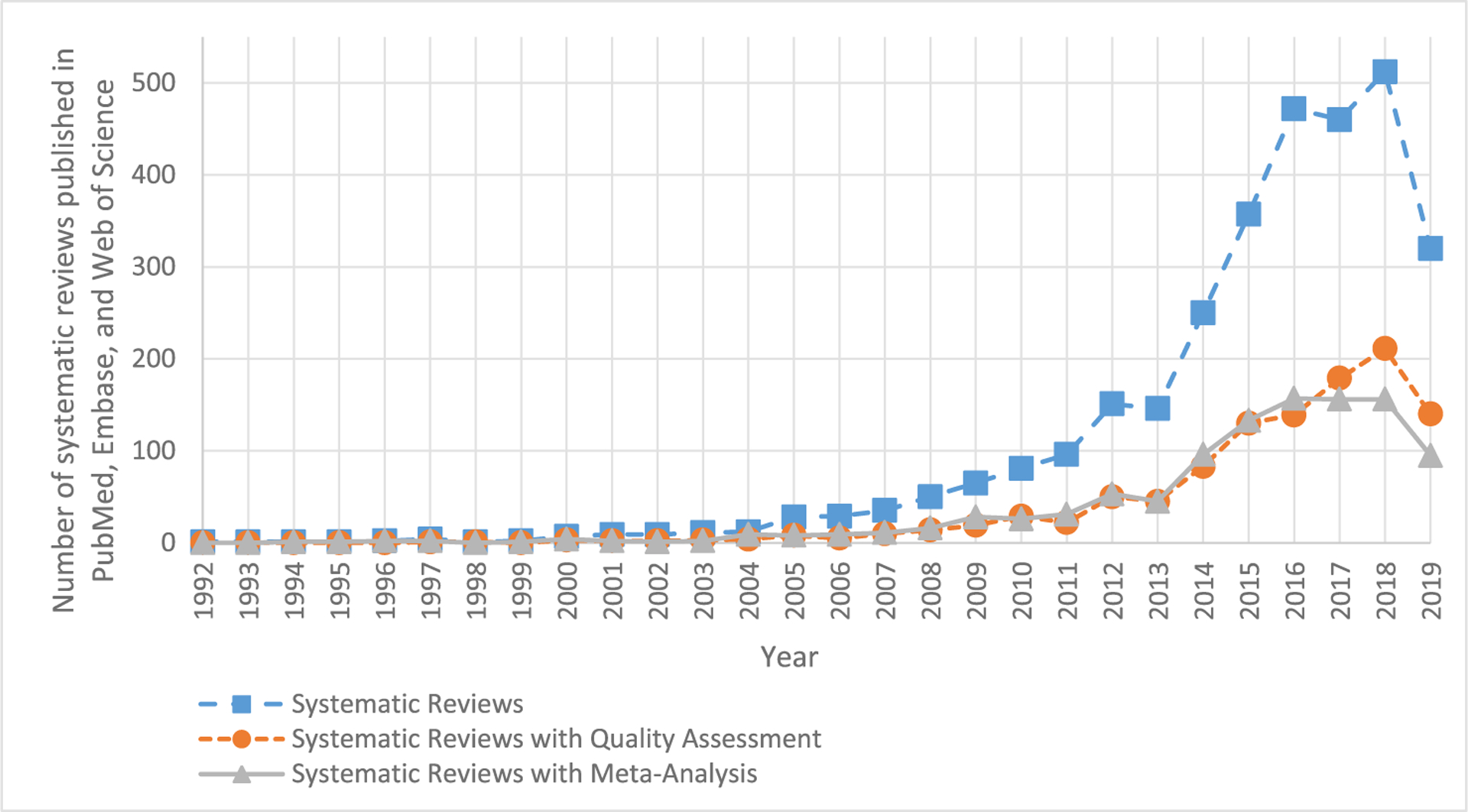
Annual frequencies of systematic reviews (SRs) of animal studies with and without quality assessment (QA) or meta-analysis (MA). There were a total of 520 SRs with both QA and MA identified in the animal SR database through 2019. Note the 2019 data represent a partial year because the database was updated in June of 2019.

Fig. 2 presents the number of animal study SRs and the trends observed over time (2018 was the last full year of data). The first SR of animal studies, an overview of the literature on placental perfusion, was published in 1992 in the *Journal of Pharmacological and Toxicological Methods*. It included studies of animal models and studies in humans (Omarini et al., 1992). Beginning in 2005, the number of SRs in animal studies increased slowly but steadily to a total of 512 SRs published in 2018.

The first MA of animal studies was published in 1994 and it investigated the effects of dietary fat intake upon mammary tumor development (Freedman, 1994). In total, 34% (n = 1045) of the reviews in the database included a MA. From 2010 to 2018, the percentage of SRs with a MA ranged from 30% to 38%, respectively.

About one-third of all SRs in the database (35%, n = 1100) include some sort of quality assessment (QA) of the studies included in the SR. The first SR of animal studies was published in 1997 and it evaluated the physiologic mechanisms in response to anemia (Hebert et al., 1997). The first formal tool that was specifically designed to assess the risk of bias (study quality) in laboratory animal studies was published in 2014 (Hooijmans et al., 2014b). The percentage SRs with QA increased slightly over time, from 36% (n = 29) in 2010 to 41% (n = 211) in 2018 and 44% in 2019 (n = 140; note that the numbers from 2019 represent 6 months as the literature search is through June 2019; Fig. 2).

The first SR of animal studies with both MA and QA was published in 2000; it was a diagnostic test SR on the accuracy of dimercaptosuccinic acid scintigraphy for the diagnosis of acute pyelonephritis (Craig et al., 2000). From 1992 to 2018, 16% (n = 458) of the SRs included both MA and QA (data not shown). In 2018, the percentage of SRs that included both MA and QA was 17% (n = 85).

### 3.4. Sources searched in systematic reviews of animal studies

The number of information sources in which the authors of the SRs searched for studies varied from 1 to 31. Examples of information sources included electronic web-based databases, such as PubMed, and the bibliographies of included studies. Seventeen percent of the reviewers used only one source, 20% used two sources, 44% used three to five sources, and 19% used more than five. PubMed (MEDLINE) was used most often (69% of the SRs), followed by Embase (34%), and Web of Science (25%).

## 4. Discussion

This paper summarizes the development of a database of animal study SRs to 1) avoid duplication of effort and, thus, reduce research waste, 2) facilitate researchers in easily identifying all systematic reviews on a specific topic, 3) aid in the creation of evidence maps, 4) serve as a resource for further analysis to advance the methodology in evidence synthesis of animal-based research.

The process of advancing methods typically begins by identifying the need for better methods. We used this database to examine meta-analytical approaches to estimating effect size in SRs of animal studies as described in a separate publication in this issue of regulatory toxicology and pharmacology (Hooijmans et al., 2020); see further details in discussion below. The database is freely available at www.data.mendeley.com (https://data.mendeley.com/drafts/6fr3nw5mpc), so researchers can use it as an efficient resource for additional analyses; for example, researchers can use the database to examine variability in meta-analysis approaches or identify the need for advancement in methods.

The database consists of all published SRs of animal studies, from the first SR published in 1992 to through June 2019. Nearly one-third (30%) of the SRs published in 2018 included at least one MA and over a third (41%) conducted a methodological quality assessment (QA). In both the overall database and in 2018 alone, approximately 17% of studies included both a MA and a QA. Given that including an assessment of methodological quality is mandatory for high-quality SRs, this analysis suggests ample room for improvement. Of note, the registration of SR protocols is currently considered good research practice, yet the registration of animal SRs lags behind that of the SRs of human studies. As of September 12, 2019, only 278 of 56,710 protocols registered in PROS-PERO are animal SR protocols, and this number is far lower than the total number SRs of animal studies published from 2017 to 2019.

Additional research into the type of methodological quality tools available and the rationale for their selection would be an appropriate next step to improve methodological quality assessment in animal study SRs. In the current version of the database it is only assessed whether or not a quality assessment was conducted or not. The type of assessment (e.g reporting quality or risk of bias assessment and the criteria used would be very interesting for future research. Moreover, a detailed analysis of the methodological quality of the included studies could indicate how to improve study design of future research using experimental animal models to generate high-quality evidence. Also, an assessment of the reporting quality of a subset of the SRs in the database could guide further implementation of SR reporting standards, such as PRISMA and ROSES, or raise awareness about adequate reporting among SR authors (Moher et al., 2009; Haddaway et al., 2018).

One research question that can be addressed by using the database is what methods researchers use when performing meta-analyses of animal studies. A detailed analysis of the SR database addressed the use of different types of effect sizes (mean difference, standardized mean difference, and normalized mean difference) and the association with heterogeneity and statistical significance. For this analysis, additional data extraction regarding the details of the meta analyses was performed on a subset of the records included in the database, namely SRs with MAs. The expanded data extraction was conducted outside of the database and the results of this analysis are presented elsewhere in this issue of regulatory toxicology and pharmacology (Hooijmans et al., 2020).

Another research topic that can be addressed by using the database is how animal studies are translated for human relevance. To this end, the database can be used to identify (via topic search) SRs in which both animal and human studies are included. The database also offers opportunities to explore recent developments in the field (e.g., methods to develop mechanistic SRs, methods to integrate evidence streams). For these topics, the database can be utilized to find compelling examples for theoretical considerations and to generate new ideas.

The database was developed with the aim for it to be open access, to enable researchers to answer meta-epidemiological questions, easily identify systematic evidence regarding a topic of interest (e.g. chemical or intervention) and to create evidence maps. As increasingly more SRs are published, updating the database is of utmost importance. The database, including a short guide on how to update the database, is freely available on www.data.Mendeley.com. Updating the database and performing more detailed data extraction are beyond the scope of the current effort.

The database currently includes bibliographical information of all included SRs and the baseline data as presented in this paper. For users of the database with more specific research questions, the search features of the database provide users with a platform (via the search menu) to identify and select SRs with a particular characteristic and export their retrieved records to Microsoft Word or Microsoft Excel. From there, users may perform additional data extraction to meet their research needs.

## 5. Conclusion

This paper presents the first database to contain all SRs of laboratory animal studies published in PubMed, Embase, and Web of Science and outlines the methods for the development. The database provides a comprehensive source that can be freely used by all researchers to easily identify all SRs on a specific topic, to avoid duplication of effort and reduce research waste, to aid in the creation of evidence maps and to explore and advance the current methodology applied to SRs of animal studies.

## Figures and Tables

**Figure C-1:**
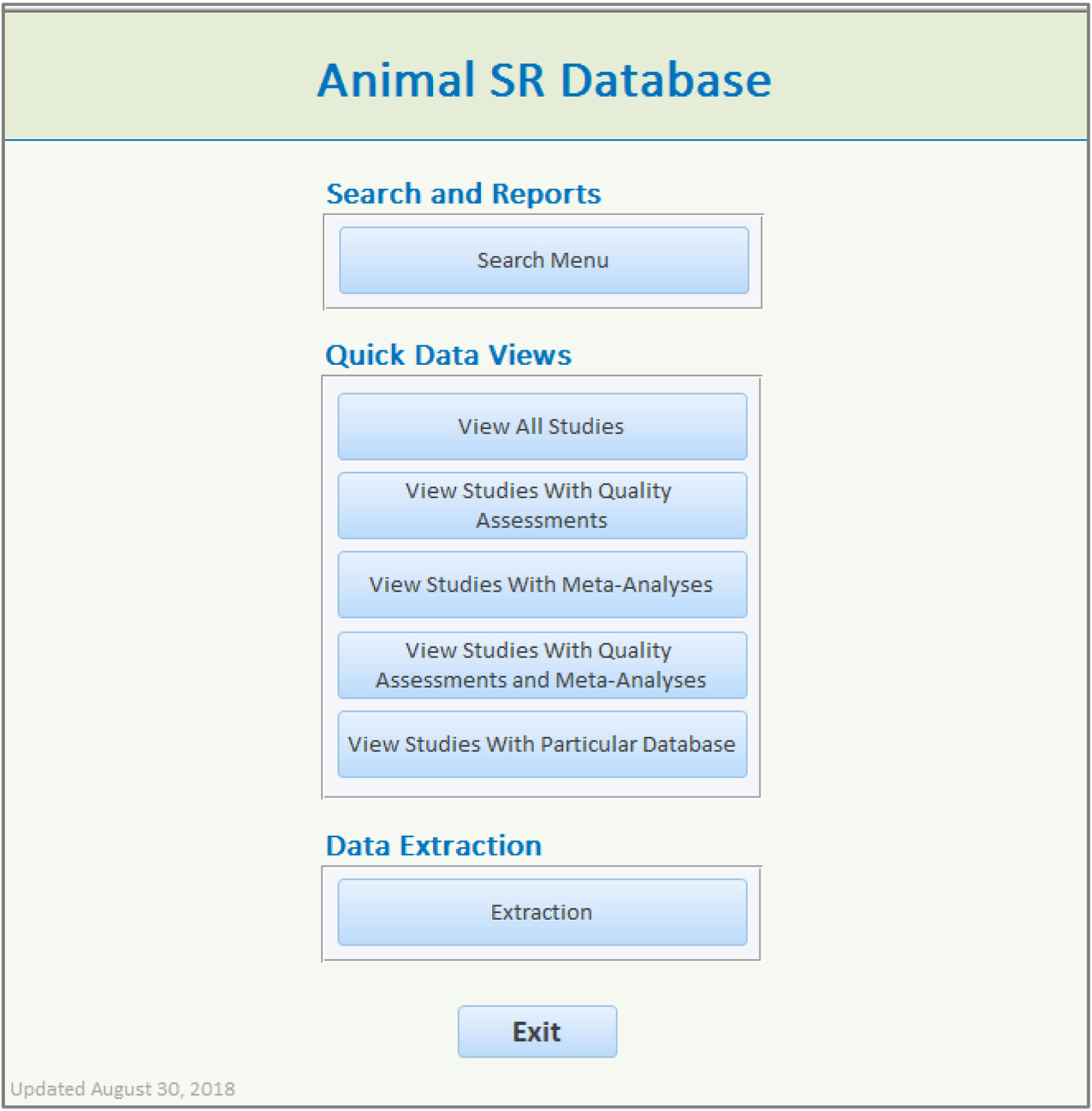
Database landing page and data view options.

**Figure C-2.**
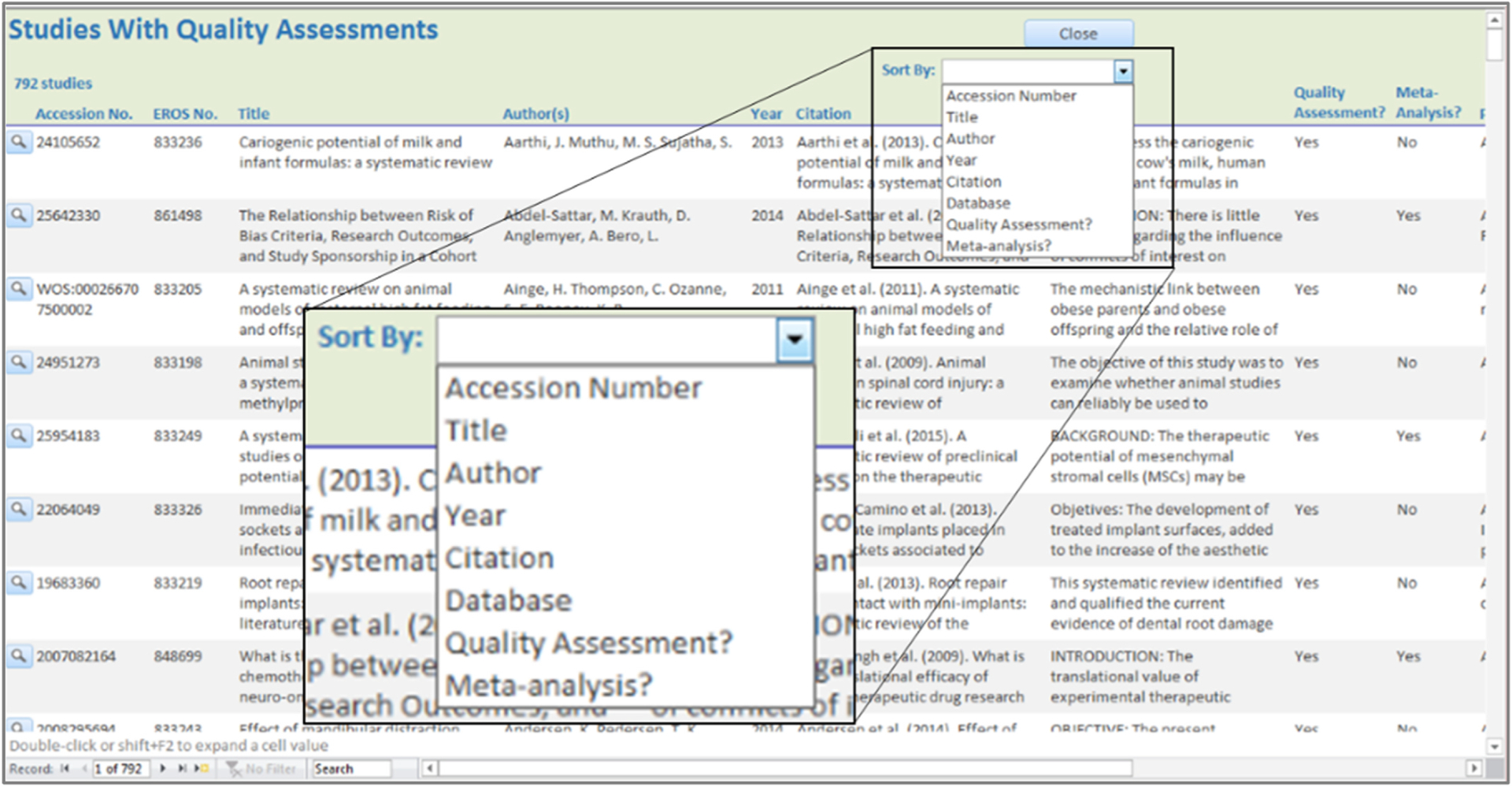
Quick Data View of studies in Microsoft Access with sort capabilities.

**Figure C-3.**
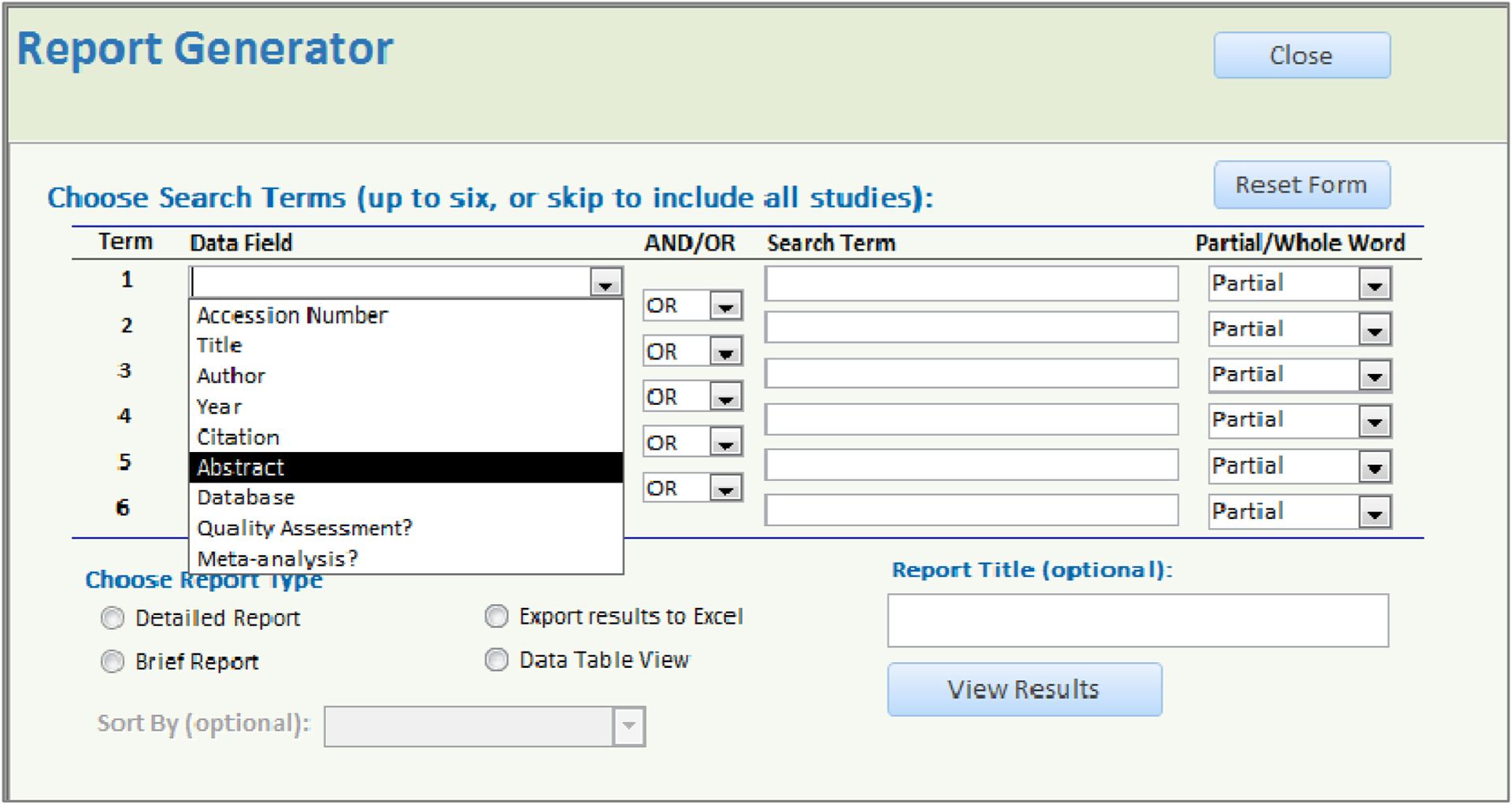
Report generator allows for keyword searches and data export.

**Box C-1.**
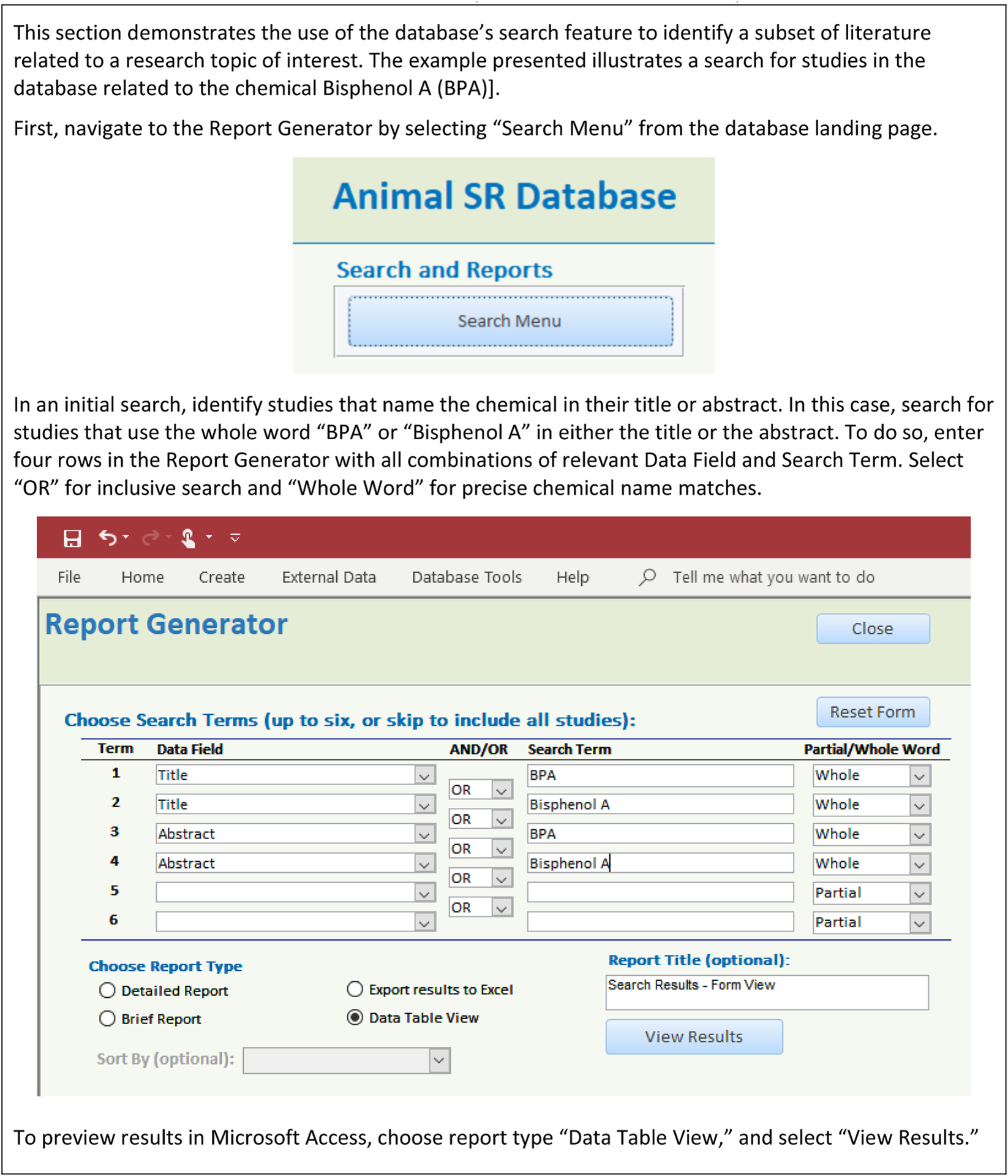

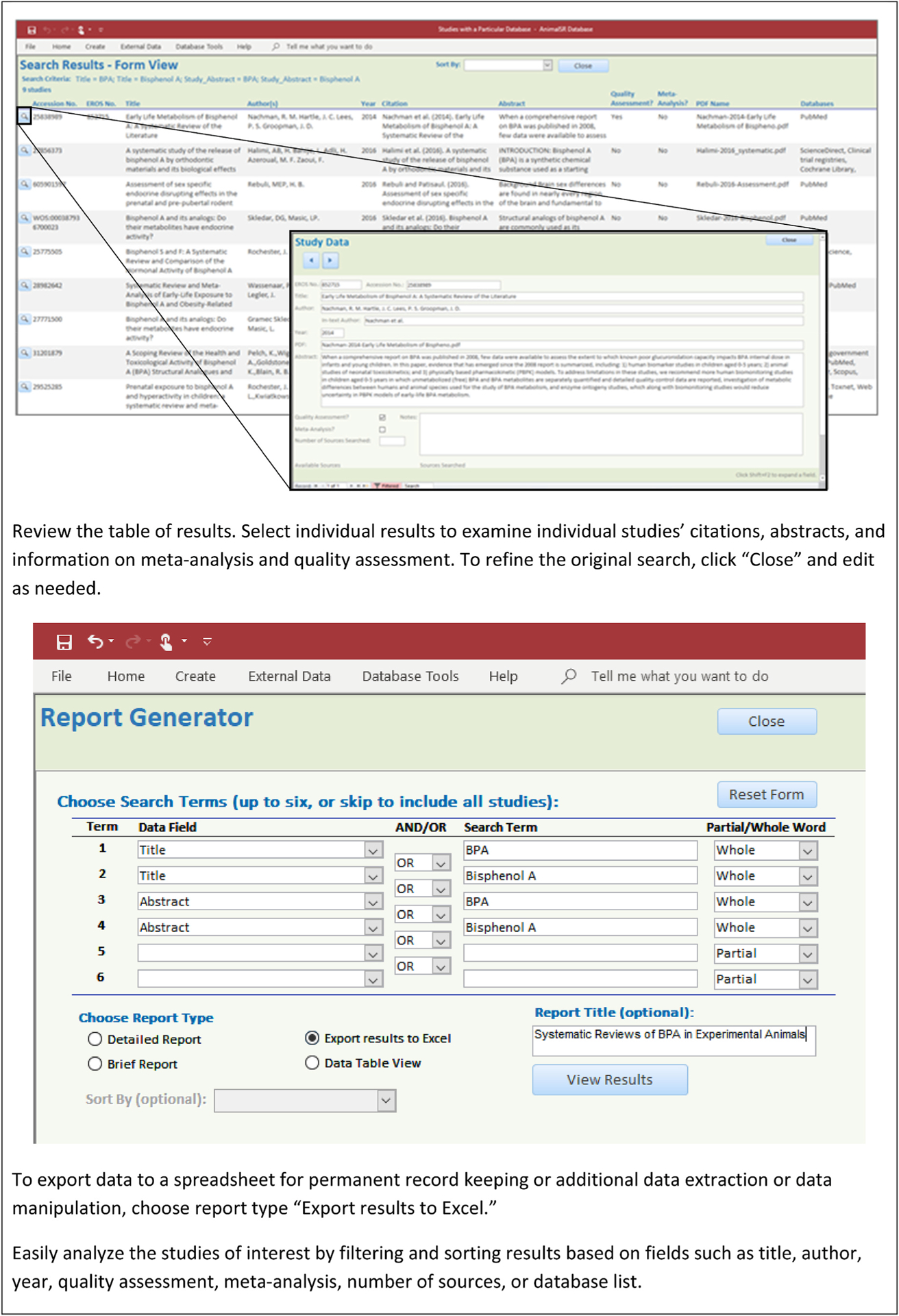
Search Feature Demonstration: Identifying Studies Related to a Specific Chemical.

## Footnotes

1 http://www.dcn.ed.ac.uk/camarades/.

2 https://www.radboudumc.nl/en/research/departments/health-evidence/systematic-review-center-for-laboratory-animal-experimentation.

3 https://www.crd.york.ac.uk/prospero/.

## Appendix A. Search Strategies

### Search strategy for PubMed Table A 1

Date of search: December 22, 2015; update search: January 10, 2017; update search: February 13, 2018; update search: June 18, 2019.

**Table A 1:** Search Strategy for Systematic Reviews of Animal Studies in the PubMed Database

Set	Search Strategy for PubMed	Notes
1	(systematic review[tiab] OR systematic reviews[tiab] OR meta-analyses[tiab] OR meta-analysis[tiab] OR metaanalyses [tiab] OR metaanalysis[tiab] OR systematic literature review[tiab] OR comprehensive literature review[tiab] OR Set Search Strategy for PubMed Systematic survey[tiab] OR systematic overview[tiab] OR “Syst Rev”[Journal] OR meta-analysis[pt] OR Systematically review[tiab] OR Systematically searched[tiab] OR Systematic search[tiab] OR systematic-literature-search*[tiab] OR Meta synthesis[tiab] OR PRISMA[tiab] OR ((electronic-database*[tiab] OR databases-search*[tiab] OR electronic-search*[tiab] OR comprehensive-search*[tiab] OR literature review[tiab] OR literature search[tiab] OR literature searches[tiab] OR literature searching[tiab] OR data collection[tiab]) AND (Pubmed[tiab] OR Medline[tiab] OR Embase [tiab] OR study-selection[tiab] OR selection-criteri*[tiab] OR Web of Science[tiab] OR Google[tiab] OR Scopus[tiab] OR BIOSIS[tiab]))) NOT (letter[pt] OR newspaper article[pt] OR comment[pt])	Apply Filters: subset Animals-SYRCLE, mentioned below *Filter your results*

Search results for Set 1 were filtered for animal studies using the following search terms.

(“animal experimentation”[MeSH Terms] OR “models, animal”[MeSH Terms] OR “invertebrates”[MeSH Terms] OR “Animals”[Mesh:noexp] OR “animal population groups”[MeSH Terms] OR “chordata”[MeSH Terms:noexp] OR “chordata, nonvertebrate”[MeSH Terms] OR “vertebrates”[MeSH Terms:noexp] OR “amphibians”[MeSH Terms] OR “birds”[MeSH Terms] OR “fishes”[MeSH Terms] OR “reptiles”[MeSH Terms] OR “mammals”[-MeSH Terms:noexp] OR “primates”[MeSH Terms:noexp] OR “artiodactyla”[MeSH Terms] OR “carnivora”[MeSH Terms] OR “cetacea”[MeSH Terms] OR “chiroptera”[MeSH Terms] OR “elephants”[MeSH Terms] OR “hyraxes”[MeSH Terms] OR “insectivora”[MeSH Terms] OR “lagomorpha”[MeSH Terms] OR “marsupialia”[MeSH Terms] OR “monotremata”[MeSH Terms] OR “perissodactyla”[MeSH Terms] OR “rodentia”[MeSH Terms] OR “scandentia”[MeSH Terms] OR “sirenia”[MeSH Terms] OR “xenarthra”[MeSH Terms] OR “haplorhini”[MeSH Terms:noexp] OR “strepsirhini”[MeSH Terms] OR “platyrrhini”[MeSH Terms] OR “tarsii”[MeSH Terms] OR “catarrhini”[MeSH Terms:noexp] OR “cercopithecidae”[MeSH Terms] OR “hylobatidae”[MeSH Terms] OR “hominidae”[MeSH Terms:noexp] OR “gorilla gorilla”[MeSH Terms] OR “pan paniscus”[MeSH Terms] OR “pan troglodytes”[MeSH Terms] OR “pongo pygmaeus”[MeSH Terms]) OR ((animals[tiab] OR animal[tiab] OR mice[Tiab] OR mus[Tiab] OR mouse[Tiab] OR murine[Tiab] OR woodmouse[tiab] OR rats[Tiab] OR rat[Tiab] OR murinae[Tiab] OR muridae[Tiab] OR cottonrat[tiab] OR cottonrats[tiab] OR hamster[tiab] OR hamsters[tiab] OR cricetinae[tiab] OR rodentia[Tiab] OR rodent[Tiab] OR rodents[Tiab] OR pigs[Tiab] OR pig[Tiab] OR swine [tiab] OR swines[tiab] OR piglets[tiab] OR piglet[tiab] OR boar[tiab] OR boars[tiab] OR “sus scrofa”[tiab] OR ferrets[tiab] OR ferret[tiab] OR polecat[tiab] OR polecats[tiab] OR “mustela putorius”[tiab] OR “guinea pigs”[Tiab] OR “guinea pig”[Tiab] OR cavia[Tiab] OR callithrix[Tiab] OR marmoset[Tiab] OR marmosets[Tiab] OR cebuella[Tiab] OR hapale[Tiab] OR octodon[Tiab] OR chinchilla[Tiab] OR chinchillas[Tiab] OR gerbil-linae[Tiab] OR gerbil[Tiab] OR gerbils[Tiab] OR jird[Tiab] OR jirds[Tiab] OR merione[Tiab] OR meriones[Tiab] OR rabbits[Tiab] OR rabbit[Tiab] OR hares[Tiab] OR hare[Tiab] OR diptera[Tiab] OR flies[Tiab] OR fly[Tiab] OR dipteral[Tiab] OR drosophila[Tiab] OR drosophilidae[Tiab] OR cats [Tiab] OR cat[Tiab] OR carus[Tiab] OR felis[Tiab] OR nematoda[Tiab] OR nematode[Tiab] OR nematodes[Tiab] OR sipunculida[Tiab] OR dogs [Tiab] OR dog[Tiab] OR canine[Tiab] OR canines[Tiab] OR canis[Tiab] OR sheep[Tiab] OR sheeps[Tiab] OR mouflon[Tiab] OR mouflons[Tiab] OR ovis[Tiab] OR goats[Tiab] OR goat[Tiab] OR capra[Tiab] OR capras[Tiab] OR rupicapra[Tiab] OR rupicapras[Tiab] OR chamois[Tiab] OR haplorhini [Tiab] OR monkey[Tiab] OR monkeys[Tiab] OR anthropoidea[Tiab] OR anthropoids[Tiab] OR saguinus[Tiab] OR tamarin[Tiab] OR tamarins[Tiab] OR leontopithecus[Tiab] OR hominidae[Tiab] OR ape[Tiab] OR apes[Tiab] OR “pan paniscus”[Tiab] OR bonobo[Tiab] OR bonobos[Tiab] OR “pan troglodytes”[Tiab] OR gibbon[Tiab] OR gibbons[Tiab] OR siamang[Tiab] OR siamangs[Tiab] OR nomascus[Tiab] OR symphalangus[Tiab] OR chimpanzee[Tiab] OR chimpanzees[Tiab] OR prosimian[Tiab] OR prosimians[Tiab] OR “bush baby”[Tiab] OR bush babies[Tiab] OR galagos[Tiab] OR galago[Tiab] OR pongidae[Tiab] OR gorilla[Tiab] OR gorillas[Tiab] OR “pongo pygmaeus”[Tiab] OR orangutan[Tiab] OR orangutans[Tiab] OR lemur[Tiab] OR lemurs[Tiab] OR lemuridae[Tiab] OR horse[Tiab] OR horses[Tiab] OR equus[Tiab] OR cow[Tiab] OR calf[Tiab] OR bull[Tiab] OR chicken[Tiab] OR chickens[Tiab] OR gallus[Tiab] OR quail[Tiab] OR bird[Tiab] OR birds[Tiab] OR quails[Tiab] OR poultry[Tiab] OR poultries [Tiab] OR fowl[Tiab] OR fowls[Tiab] OR reptile[Tiab] OR reptilia[Tiab] OR reptiles[Tiab] OR snakes[Tiab] OR snake[Tiab] OR lizard[Tiab] OR lizards[Tiab] OR alligator[Tiab] OR alligators[Tiab] OR crocodile[Tiab] OR crocodiles[Tiab] OR turtle[Tiab] OR turtles[Tiab] OR amphibian[Tiab] OR amphibians[Tiab] OR amphibia[Tiab] OR frog[Tiab] OR frogs[Tiab] OR bombina[Tiab] OR salientia[Tiab] OR toad[Tiab] OR toads[Tiab] OR “epidalea calamita”[Tiab] OR salamander[Tiab] OR salamanders[Tiab] OR eel[Tiab] OR eels[Tiab] OR fish[Tiab] OR fishes[Tiab] OR pisces[Tiab] OR catfish[Tiab] OR catfishes[Tiab] OR siluriformes[Tiab] OR arius[Tiab] OR heteropneustes[Tiab] OR sheatfish[Tiab] OR perch[Tiab] OR perches [Tiab] OR percidae[Tiab] OR perca[Tiab] OR trout[Tiab] OR trouts[Tiab] OR char[Tiab] OR chars[Tiab] OR salvelinus[Tiab] OR minnow[Tiab] OR cyprinidae[Tiab] OR carps[Tiab] OR carp[Tiab] OR zebrafish[Tiab] OR zebrafishes[Tiab] OR goldfish[Tiab] OR goldfishes[Tiab] OR guppy[Tiab] OR guppies[Tiab] OR chub[Tiab] OR chubs[Tiab] OR tinca[Tiab] OR barbels[Tiab] OR barbus[Tiab] OR pimephales[Tiab] OR promelas[Tiab] OR “poecilia reticulata”[Tiab] OR mullet[Tiab] OR mullets[Tiab] OR eel[Tiab] OR eels[Tiab] OR seahorse[Tiab] OR seahorses[Tiab] OR mugil curema [Tiab] OR atlantic cod[Tiab] OR shark[Tiab] OR sharks[Tiab] OR catshark[Tiab] OR anguilla[Tiab] OR salmonid[Tiab] OR salmonids[Tiab] OR whitefish[Tiab] OR whitefishes[Tiab] OR salmon[Tiab] OR salmons[Tiab] OR sole[Tiab] OR solea[Tiab] OR lamprey[Tiab] OR lampreys[Tiab] OR pumpkinseed[Tiab] OR sunfish[Tiab] OR sunfishes[Tiab] OR tilapia[Tiab] OR tilapias[Tiab] OR turbot[Tiab] OR turbots[Tiab] OR flatfish[Tiab] OR flatfishes[Tiab] OR sciuridae[Tiab] OR squirrel[Tiab] OR squirrels[Tiab] OR chipmunk[Tiab] OR chipmunks[Tiab] OR suslik[Tiab] OR susliks[Tiab] OR vole[Tiab] OR voles[Tiab] OR lemming[Tiab] OR lemmings[Tiab] OR muskrat[Tiab] OR muskrats[Tiab] OR lemmus[Tiab] OR otter[Tiab] OR otters[Tiab] OR marten[Tiab] OR martens[Tiab] OR martes[Tiab] OR weasel[Tiab] OR badger[Tiab] OR badgers[Tiab] OR ermine[Tiab] OR mink [Tiab] OR minks[Tiab] OR sable[Tiab] OR sables[Tiab] OR gulo[Tiab] OR gulos[Tiab] OR wolverine[Tiab] OR wolverines[Tiab] OR mustela[Tiab] OR llama[Tiab] OR llamas[Tiab] OR alpaca[Tiab] OR alpacas[Tiab] OR camelid[Tiab] OR camelids[Tiab] OR guanaco[Tiab] OR guanacos[Tiab] OR chiroptera[Tiab] OR chiropteras[Tiab] OR bat[Tiab] OR bats[Tiab] OR fox[Tiab] OR foxes[Tiab] OR iguana[Tiab] OR iguanas[Tiab] OR xenopus laevis[Tiab] OR parakeet[Tiab] OR parakeets[Tiab] OR parrot[Tiab] OR parrots[Tiab] OR donkey[Tiab] OR donkeys[Tiab] OR mule[Tiab] OR mules [Tiab] OR zebra[Tiab] OR zebras[Tiab] OR shrew[Tiab] OR shrews[Tiab] OR bison[Tiab] OR bisons[Tiab] OR buffalo[Tiab] OR buffaloes[Tiab] OR deer[Tiab] OR deers[Tiab] OR bear[Tiab] OR bears[Tiab] OR panda[Tiab] OR pandas[Tiab] OR “wild hog”[Tiab] OR “wild boar”[Tiab] OR fitchew [Tiab] OR fitch[Tiab] OR beaver[Tiab] OR beavers[Tiab] OR jerboa[Tiab] OR jerboas[Tiab] OR capybara[Tiab] OR capybara.

### Search strategy for embase Table A 2

Date of search: December 28, 2015; search update: January 10, 2017; update search: February 13, 2018; update search: June 18, 2019.

**Table A 2:** Search Strategy for Systematic Reviews of Animal Studies in the Embase Database

Set	Search Strategy for Embase	Notes
1	‘Animal experiment’/exp OR ‘animal model’/exp OR ‘experimental animal’/exp OR ‘transgenic animal’/exp OR ‘male animal’/exp OR ‘female animal’/exp OR ‘juvenile animal’/exp OR animal OR chordata OR vertebrate OR tetrapod OR fish/exp OR amniote OR amphibia/exp OR mammal OR reptile/exp OR sauropsid/exp OR therian OR monotremate/exp OR ‘placental mammals’ OR marsupial/exp OR Euarchontoglires OR Afrotheria/exp OR Boreoeutheria/exp OR Laurasiatheria/exp OR Xenarthra/exp OR primate/exp OR Dermoptera/exp OR Glires/exp OR Scandentia/exp OR Haplorhini OR prosimian/exp OR simian OR tarsiiform/exp OR Catarrhini OR Platyrrhini/exp OR ape OR Cercopithecidae/exp OR hominid OR hylobatidae/exp OR chimpanzee/exp OR gorilla/exp OR orangutan/exp	
2	(animal OR animals OR pisces OR fish OR fishes OR catfish OR catfishes OR sheatfish OR silurus OR arius OR heteropneustes OR clarias OR gariepinus OR “fathead minnow” OR “fathead minnows” OR pimephales OR promelas OR cichlidae OR trout OR trouts OR char OR chars OR salvelinus OR salmo OR oncorhynchus OR guppy OR guppies OR millionfish OR poecilia OR goldfish OR goldfishes OR carassius OR auratus OR mullet OR mullets OR mugil OR curema OR shark OR sharks OR cod OR cods OR gadus OR morhua OR carp OR carps OR cyprinus OR carpio OR killifish OR eel OR eels OR anguilla OR zander OR sander OR lucioperca OR stizostedion OR turbot OR turbots OR psetta OR flatfish OR flatfishes OR plaice OR pleuronectes OR platessa OR tilapia OR tilapias OR oreochromis OR sarotherodon OR “common sole” OR “dover sole” OR solea OR zebrafish OR zebrafishes OR danio OR rerio OR seabass OR dicentrarchus OR labrax OR morone OR lamprey OR lampreys OR petromyzon OR pumpkinseed OR pumpkinseeds OR lepomis OR gibbosus OR herring OR clupea OR harengus OR amphibia OR amphibian OR amphibians OR anura OR salientia OR frog OR frogs OR rana OR toad OR toads OR bufo OR xenopus OR laevis OR bombina OR epidalea OR calamita OR salamander OR salamanders OR newt OR newts OR triturus OR reptilia OR reptile OR reptiles OR “bearded dragon” OR pogona OR vitticeps OR iguana OR iguanas OR lizard OR lizards OR “anguis fragilis” OR turtle OR turtles OR snakes OR snake OR aves OR bird OR birds OR quail OR quails OR coturnix OR bobwhite OR colinus OR virginianus OR poultry OR poultries OR fowl OR fowls OR chicken OR chickens OR gallus OR “zebra finch” OR taeniopygia OR guttata OR canary OR canaries OR serinus OR canaria OR parakeet OR parakeets OR grasskeet OR parrot OR parrots OR psittacine OR psittacines OR shelduck OR tadorna OR goose OR geese OR branta OR leucopsis OR woodlark OR lullula OR flycatcher OR ficedula OR hypoleuca OR dove OR doves OR geopelia OR cuneata OR duck OR ducks OR greylag OR graylag OR anser OR harrier OR “circus pygargus” OR “red knot” OR “great knot” OR calidris OR canutus OR godwit OR limosa OR lapponica OR meleagris OR gallopavo OR jackdaw OR corvus OR monedula OR ruff OR philomachus OR pugnax OR lapwing OR peewit OR plover OR vanellus OR swan OR cygnus OR columbianus OR bewickii OR gull OR chroicocephalus OR ridibundus OR albifrons OR “great tit” OR parus OR aythya OR fuligula OR streptopelia OR risoria OR spoonbill OR platalea OR leucorodia OR blackbird OR turdus OR merula OR “blue tit” OR cyanistes OR pigeon OR pigeons OR columba OR pintail OR anas OR starling OR sturnus OR owl OR “athene noctua” OR pochard OR ferina OR cockatiel OR nymphicus OR hollandicus OR skylark OR alauda OR tern OR sterna OR teal OR crecca OR oystercatcher OR haematopus OR ostralegus OR shrew OR shrews OR sorex OR araneus OR crocidura OR russula OR “european mole” OR talpa OR chiroptera OR bat OR bats OR eptesicus OR serotinus OR myotis OR dasycneme OR daubentonii OR pipistrelle OR pipistrellus OR cat OR cats OR felis OR catus OR feline OR dog OR dogs OR canis OR canine OR canines OR otter OR otters OR lutra OR badger OR badgers OR meles OR fitchew OR fitch OR foumart OR foulmart OR ferrets OR ferret OR polecat OR polecats OR mustela OR putorius OR weasel OR weasels OR fox OR foxes OR vulpes OR “common seal” OR phoca OR vitulina OR “grey seal” OR halichoerus OR horse OR horses OR equus OR equine OR equidae OR donkey OR donkeys OR mule OR mules OR pig OR pigs OR swine OR swines OR hog OR hogs OR boar OR boars OR porcine OR piglet OR piglets OR sus OR scrofa OR llama OR llamas OR lama OR glama OR deer OR deers OR cervus OR elaphus OR cow OR cows OR “bos Taurus” OR “bos indicus” OR bovine OR bull OR bulls OR cattle OR bison OR bisons OR sheep OR sheeps OR “ovis aries” OR ovine OR lamb OR lambs OR mouflon OR mouflons OR goat OR goats OR capra OR caprine OR chamois OR rupicapra OR leporidae OR lagomorpha OR lagomorph OR rabbit OR rabbits OR oryctolagus OR cuniculus OR laprine OR hares OR lepus OR rodentia OR rodent OR rodents OR murinae OR mouse OR mice OR mus OR musculus OR murine OR woodmouse):ti,ab	
3	(apodemus OR rat OR rats OR rattus OR norvegicus OR “guinea pig” OR “guinea pigs” OR cavia OR porcellus OR hamster OR hamsters OR mesocricetus OR cricetulus OR cricetus OR gerbil OR gerbils OR jird OR jirds OR meriones OR unguiculatus OR jerboa OR jerboas OR jaculus OR chinchilla OR chinchillas OR beaver OR beavers OR “castor fiber” OR “castor Canadensis” OR sciuridae OR squirrel OR squirrels OR sciurus OR chipmunk OR chipmunks OR marmot OR marmots OR marmota OR suslik OR susliks OR spermophilus OR cynomys OR cottonrat OR cottonrats OR sigmodon OR vole OR voles OR microtus OR myodes OR glareolus OR primate OR primates OR prosimian OR prosimians OR lemur OR lemurs OR lemuridae OR loris OR “bush baby” OR “bush babies” OR bushbaby OR bushbabies OR galago OR galagos OR anthropoidea OR anthropoids OR simian OR simians OR monkey OR monkeys OR marmoset OR marmosets OR callithrix OR cebuella OR tamarin OR tamarins OR saguinus OR leontopithecus OR “squirrel monkey” OR “squirrel monkeys” OR saimiri OR “night monkey” OR “night monkeys” OR “owl	
	monkey” OR “owl monkeys” OR douroucoulis OR aotus OR “spider monkey” OR “spider monkeys” OR ateles OR baboon OR baboons OR papio OR “rhesus monkey” OR macaque OR macaca OR mulatta OR cynomolgus OR fascicularis OR “green monkey” OR “green monkeys” OR chlorocebus OR vervet OR vervets OR pygerythrus OR hominoidea OR ape OR apes OR hylobatidae OR gibbon OR gibbons OR siamang OR siamangs OR nomascus OR symphalangus OR hominidae OR orangutan OR orangutans OR pongo OR chimpanzee OR chimpanzees OR “pan troglodytes” OR bonobo OR bonobos OR “pan paniscus” OR gorilla OR gorillas OR troglodytes):ti,ab	
4	1 OR 2 OR 3	
5	limit set 4 to (“meta analysis” OR “systematic review”)	
6	(“systematic review” OR “meta-analysis” OR metaanalysis):ti OR (((meta-analyses OR meta-analysis OR metaanalyses OR metaanalysis OR “systematic overview”):ti,ab,de OR “systematic reviews”:jt OR “meta analysis”:jt OR “Meta synthesis”:ti,ab,de OR (Systematic* adj2 (Review OR literature OR Reviews OR survey OR search*)):ti,ab,de) and (“Data collection” OR “Data extraction” OR “Inclusion Criteria” OR “Exclusion criteria” OR Search* OR Literature OR Pubmed OR Medline OR Embase OR selection OR Web of Science OR Google OR Scopus OR BIOSIS):ti,ab,de)	Limited to study types: NonHuman, Meta analysis, Systematic review, meta analysis (topic), systematic review (topic). The topic options are not available during the search 5.
7	4 AND 6	
8	5 OR 7	
9	Limit set 8 to (book or book series or conference abstract or conference paper or conference proceeding or editorial or letter)	
10	8 NOT 9	Limited to SRs or MAs (see Table A2, row 6 for actual Embase topics used)

### Search strategy for web of science Table A 3

Date of search: December 22, 2015; 2017 update: January 10, 2017; update search: February 13, 2018; update search: June 18, 2019.

**Table A 3:** Search Strategy for Systematic Reviews of Animal Studies in the Web of Science Database

Set	Search Strategy for Web of Science	Notes
1	**TOPIC:** ((“systematic review” OR “systematic reviews” OR “meta-analyses” OR “meta-analysis” OR “metaanalyses” OR “metaanalysis” OR “systematic literature review” OR “Systematic survey”[tiab] OR “systematic overview” OR “Systematically review” OR “Systematically searched” OR “Systematic search” OR “Meta synthesis”)) Indexes = SCI-EXPANDED, SSCI, A&HCI, ESCI Timespan = All years	
2	**TOPIC:** ((“literature search” OR “literature searches” OR “literature searching” OR “data collection” OR “electronic-database*” OR “databases-search*” OR “electronic-search*” OR “comprehensive-search*” OR “literature search” OR “literature searches” OR “literature searching” OR “data collection”) AND (Pubmed OR “Medline” OR “Embase” OR “selection” OR “Web of Science” OR “Google” OR “Scopus” OR BIOSIS)) Indexes = SCI-EXPANDED, SSCI, A&HCI, ESCI Timespan = All years	
3	1 OR 2 Indexes = SCI-EXPANDED, SSCI, A&HCI, ESCI Timespan = All years	
4	**TOPIC:** ((animal OR animals OR pisces OR fish OR fishes OR catfish OR catfishes OR sheatfish OR silurus OR arius OR heteropneustes OR clarias OR gariepinus OR “fathead minnow” OR “fathead minnows” OR pimephales OR promelas OR cichlidae OR trout OR trouts OR char OR chars OR salvelinus OR salmo OR oncorhynchus OR guppy OR guppies OR millionfish OR poecilia OR goldfish OR goldfishes OR carassius OR auratus OR mullet OR mullets OR mugil OR curema OR shark OR sharks OR cod OR cods OR gadus OR morhua OR carp OR carps OR cyprinus OR carpio OR killifish OR eel OR eels OR anguilla OR zander OR sander OR lucioperca OR stizostedion OR turbot OR turbots OR psetta OR flatfish OR flatfishes OR plaice OR pleuronectes OR platessa OR tilapia OR tilapias OR oreochromis OR sarotherodon OR “common sole” OR “dover sole” OR solea OR zebrafish OR zebrafishes OR danio OR rerio OR seabass OR dicentrarchus OR labrax OR morone OR lamprey OR lampreys OR petromyzon OR pumpkinseed OR pumpkinseeds OR lepomis OR gibbosus OR herring OR clupea OR harengus OR amphibia OR amphibian OR amphibians OR anura OR salientia OR frog OR frogs OR rana OR toad OR toads OR bufo OR xenopus OR laevis OR bombina OR epidalea OR calamita OR salamander OR salamanders OR newt OR newts OR triturus OR reptilia OR reptile OR reptiles OR “bearded dragon” OR pogona OR vitticeps OR iguana OR iguanas OR lizard OR lizards OR “anguis fragilis” OR turtle OR turtles OR snakes OR snake OR aves OR bird OR birds OR quail OR quails OR coturnix OR bobwhite OR colinus OR virginianus OR poultry OR poultries OR fowl OR fowls OR chicken OR chickens OR gallus OR “zebra finch” OR taeniopygia OR guttata OR canary OR canaries OR serinus OR canaria OR parakeet OR parakeets OR grasskeet OR parrot OR parrots OR psittacine OR psittacines OR shelduck OR tadorna OR goose OR geese OR branta OR leucopsis OR woodlark OR lullula OR flycatcher OR ficedula OR hypoleuca OR dove OR doves OR geopelia OR cuneata OR duck OR ducks OR greylag OR graylag OR anser OR harrier OR “circus pygargus” OR “red knot” OR “great knot” OR calidris OR canutus OR godwit OR limosa OR lapponica OR meleagris OR gallopavo OR jackdaw OR corvus OR monedula OR ruff OR philomachus OR pugnax OR lapwing OR peewit OR plover OR vanellus OR swan OR cygnus OR columbianus OR bewickii OR gull OR chroicocephalus OR ridibundus OR albifrons OR “great tit” OR parus OR aythya OR fuligula OR streptopelia OR risoria OR spoonbill OR platalea OR leucorodia OR blackbird OR turdus OR merula OR “blue tit” OR cyanistes OR pigeon OR pigeons OR columba OR pintail OR anas OR starling OR sturnus OR owl OR “athene noctua” OR pochard OR ferina OR cockatiel OR nymphicus OR hollandicus OR skylark OR alauda OR tern OR sterna OR teal OR crecca OR oystercatcher OR haematopus OR ostralegus OR shrew OR shrews OR sorex OR araneus OR crocidura OR russula OR “european mole” OR talpa OR chiroptera OR bat OR bats OR eptesicus OR serotinus OR myotis OR dasycneme OR daubentonii OR pipistrelle OR pipistrellus OR cat OR cats OR felis OR catus OR feline OR dog OR dogs OR canis OR canine OR canines OR otter OR otters OR lutra OR badger OR badgers OR meles OR fitchew OR fitch OR foumart OR foulmart OR ferrets OR ferret OR polecat OR polecats OR mustela OR putorius OR weasel OR weasels OR fox OR foxes OR vulpes OR “common seal” OR phoca OR vitulina OR “grey seal” OR halichoerus OR horse OR horses OR equus OR equine OR equidae OR donkey OR donkeys OR mule OR mules OR pig OR pigs OR swine OR swines OR hog OR hogs OR boar OR boars OR porcine OR piglet OR piglets OR sus OR scrofa OR llama OR llamas OR lama OR glama OR deer OR deers OR cervus OR elaphus OR cow OR cows OR “bos taurus” OR “bos indicus” OR bovine OR bull OR bulls OR cattle OR bison OR bisons OR sheep OR sheeps OR “ovis aries” OR ovine OR lamb OR lambs OR mouflon OR mouflons OR goat)) Indexes = SCI-EXPANDED, SSCI, A&HCI, ESCI Timespan = All years	
5	**TOPIC:** ((goats OR capra OR caprine OR chamois OR rupicapra OR leporidae OR lagomorpha OR lagomorph OR rabbit OR rabbits OR oryctolagus OR cuniculus OR laprine OR hares OR lepus OR rodentia OR rodent OR rodents OR murinae OR mouse OR mice OR mus OR musculus OR murine OR woodmouse OR apodemus OR rat OR rats OR rattus OR norvegicus OR “guinea pig” OR “guinea pigs” OR cavia OR porcellus OR hamster OR hamsters OR mesocricetus OR cricetulus OR cricetus OR gerbil OR gerbils OR jird OR jirds OR meriones OR unguiculatus OR jerboa OR jerboas OR jaculus OR chinchilla OR chinchillas OR beaver OR beavers OR castor fiber OR “castor canadensis” OR sciuridae OR squirrel OR squirrels OR sciurus OR chipmunk OR chipmunks OR marmot OR marmots OR marmota OR suslik OR susliks OR spermophilus OR cynomys OR cottonrat OR cottonrats OR sigmodon OR vole OR voles OR microtus OR myodes OR glareolus OR primate OR primates OR prosimian OR prosimians OR lemur OR lemurs OR lemuridae OR loris OR “bush baby” OR “bush babies” OR bushbaby OR bushbabies OR galago OR galagos OR anthropoidea OR anthropoids OR simian OR simians OR monkey OR monkeys OR marmoset OR marmosets OR callithrix OR cebuella OR tamarin OR tamarins OR saguinus OR leontopithecus OR “squirrel monkey” OR “squirrel monkeys” OR saimiri OR “night monkey” OR “night monkeys” OR “owl monkey” OR “owl monkeys” OR douroucoulis OR aotus OR “spider monkey” OR “spider monkeys” OR ateles OR baboon OR baboons OR papio OR “rhesus monkey” OR macaque OR macaca OR mulatta OR cynomolgus OR fascicularis OR “green monkey” OR “green monkeys” OR chlorocebus OR vervet OR vervets OR pygerythrus OR hominoidea OR ape OR apes OR hylobatidae OR gibbon OR gibbons OR siamang OR siamangs OR nomascus OR symphalangus OR hominidae OR orangutan OR orangutans OR pongo OR chimpanzee OR chimpanzees OR “pan troglodytes” OR bonobo OR bonobos OR pan paniscus OR gorilla OR gorillas OR troglodytes)) Indexes = SCI-EXPANDED, SSCI, A&HCI, ESCI Timespan = All years	
6	4 OR 5 Indexes = SCI-EXPANDED, SSCI, A&HCI, ESCI Timespan = All years	
7	3 AND 6 Indexes = SCI-EXPANDED, SSCI, A&HCI, ESCI Timespan = All years	
8	3 AND 6	Search results filtered in R to remove references with search terms only in the keyword field^a^
	Refined by:DOCUMENT TYPES: (ARTICLE OR REVIEW) Indexes = SCI-EXPANDED, SSCI, A&HCI, ESCI Timespan = All years

a Web of Science does not allow users to search the title and abstract fields only. To search title and abstract in Web of Science, users must search by *Topic*, which includes not only title and abstract, but also the keyword field. To limit the search results requiring manual review, results from Web of Science were further filtered to remove results where keywords were only found in the keyword field (i.e., not found in title or abstract). This additional step was completed using R and was conducted on the initial search and all subsequent search updates.

## Appendix B. Study election and Data Collection Process

### Study selection

Following de-duplication of the initial literature search results in 2016, all studies with the following specific terms were automatically screened at the title-and-abstract level: systematic review, systematic literature review, meta-analysis, and meta-analysis. The remaining results of the initial literature search were then prioritized in ICF’s Document Classification and Topic Extraction Resource (DoCTER; Durham, North Carolina, USA) for manual review at the title-and-abstract level. This prioritization involved unsupervised machine learning or topic extraction followed by supervised machine learning (Varghese et al., 2018a). The unsupervised machine learning and topic extraction step grouped the references into topic areas based on automated text analysis of their titles and abstracts to produce a set of keywords for each group that served as the topic signature. The keywords were reviewed to determine the topics covered in each group and, thus, to prioritize which groups of references for review. After an initial set of references were evaluated using the title and abstract, these studies were used as a training dataset to predict which of the unscreened references were relevant (i.e., supervised machine learning). All studies that were predicted not relevant were excluded at the supervised learning stage. Remaining references were evaluated for relevance by two independent screeners. Conflicts were resolved by a third screener as necessary. Studies were considered high probability inclusion if the study was either a systematic review or meta-analysis and was conducted using laboratory animals.

All studies categorized as high probability inclusion using the title and abstract were evaluated using manual full-text screening by two independent screeners. Conflicts were resolved by a third screener as necessary. Studies were included, unless they met one or more of the following exclusion criteria: 1) aim of the paper was not a systematic review, 2) paper did not include laboratory animals, 3) paper did not state the eligibility criteria for primary studies, 4) paper did not specify what sources (e.g., databases) were searched, 5) paper did not specify the search terms used in the search, or the 6) full-text paper could not be retrieved. In the case that it was unclear whether a reference was an SR that included animal studies, the reference was included in the database.

### Data collection process

Studies identified as relevant (or possibly relevant) during full-text screening were moved forward to data into database software (a new database was developed in Microsoft Access, Redmond, Washington, USA). The following data elements (characteristics) were captured for each reference:

- Bibliographical details [title, author(s), year of publication, abstract]
- Study includes a quality assessment: yes or no
- Study includes a meta-analysis: yes or no
- Total number of sources (e.g., electronic databases) searched in the study
- Names of sources included in the study

For each study, data elements were extracted by a single person. The instructions provided to extractors regarding definitions for quality assessment, meta-analysis, and sources were the following:

- Quality assessment is when authors review studies that met the inclusion criteria to determine the quality of the study.
- Studies must have used a formal quality assessment tool or an explaination of the criteria used to evaluate the study.
- Publication bias analysis does NOT count as quality assessment.
- Level of evidence strictly based on the type of study does NOT count as quality assessment unless the study refers to a specific assessment tool that has levels.
- Meta-analysis is when data from included studies are extracted and combined in a quantitative assessment.
- Cluster analysis, R ratios, pooled risk ratio, or pooled odds ratio would be examples of meta-analyses.
- Sources of animal SRs can be:
- Electronic databases/web-based collections of literature (e.g., PubMed).
- Studies identified from the bibliographies of other studies (e.g, included studies, previously published (systematic) reviews).
- Studies known to authors (e.g., hand-picked publications that the authors had prior knowledge of).
- Specific journals may have been searched; in this case, each journal is counted as a separate source.
- Conference abstracts. These were counted as one source, even if multiple conferences are listed, and all conferences were listed in the notes field.

## Appendix C. Database Features

Database users can view all references (also called records or studies) included in the database, a subset of records based on the elements defined in the data extraction step or perform keyword searches to refine the record selection. The database includes three sections (Fig. C-1), the first two of which focus on record querying (Search and reports, and Quick Data Views). The third section relates to the data extraction (also called data collection) step when adding records to the database; it is not detailed in the current document.

### How to view all records

When working within the Microsoft Access database, the Quick Data Views section provides quick navigation to view all studies.

Users can also generate a report of all studies for viewing outside of the Microsoft Access database under the Search and Reports section. Report types include text documents for static viewing, and a Microsoft Excel export to allow for customized data sorting and manipulation. To generate a report of all records within the database, users can select the preferred report type without entering any additional search terms.

### How to view a subset of records

When working within the Microsoft Access database, the Quick Data Views section provides quick navigation to view:

- Studies with a quality assessment
- Studies with a meta-analysis
- Studies with a quality assessment *and* a meta-analysis
- Studies with a particular database. The term database was used to refer to electronic web-based databases of literature (e.g., PubMed) as well as other sources (e.g., bibliographies of included papers).

Within each of these quick views, users can sort by one of the data extraction fields (Fig. C-2) Users can apply similar sort features in the Search and Reports section.

### How to search records

This database allows for refined data viewing by searching the database for records that meet the desired criteria. Under the Search and Reports section, users can search up to six terms in nine data fields (Fig. C-3). The database supports partial and whole word searches. For example, users can search for papers relating to a particular disease model by searching for the disease in the title or abstract data fields. Users can choose between two text document views, data export in Microsoft Excel, or table viewing within Microsoft Access. Box C-1 offers a step-by-step example of how to search for an environmental chemical exposure in the database of animal studies SRs.
